# The benefits and challenges of embedding specialist palliative care teams within homeless hostels to enhance support and learning: Perspectives from palliative care teams and hostel staff

**DOI:** 10.1177/02692163211006318

**Published:** 2021-03-29

**Authors:** Megan Armstrong, Caroline Shulman, Briony Hudson, Niamh Brophy, Julian Daley, Nigel Hewett, Patrick Stone

**Affiliations:** 1 Pathway Charity, London, UK; 2 Marie Curie Palliative Care Research Department, Division of Psychiatry, University College London, London, UK; 3 Marie Curie, Vauxhall, London, UK; 4 St Ann’s Hospice, Manchester, UK

**Keywords:** Palliative care, homelessness, hostels, qualitative

## Abstract

**Background:**

People residing in UK homeless hostels experience extremely high rates of multi-morbidity, frailty and age-related conditions at a young age. However, they seldom receive palliative care with the burden of support falling to hostel staff.

**Aim:**

To evaluate a model embedding palliative specialists, trained as ‘homelessness champions’, into hostels for two half-days a month to provide support to staff and residents and facilitate a multidisciplinary approach to care.

**Design:**

An exploratory qualitative design.

**Setting/participants:**

Four homeless hostels in London, UK, including nine hostel managers/support staff and seven palliative care specialists (five nurses and two social workers).

**Results:**

Benefits to introducing the model included: developing partnership working between hostel staff and palliative care specialists, developing a holistic palliative ethos within the hostels and improving how hostel staff seek support and connect with local external services. Challenges to implementation included limited time and resources, and barriers related to primary care.

**Conclusion:**

This is the first evaluation of embedding palliative care specialists within homeless hostels. Inequity in health and social care access was highlighted with evidence of benefit of this additional support for both hostel staff and residents. Considering COVID-19, future research should explore remote ways of working including providing in-reach support to homelessness services from a range of services and organisations.

**What is already known about the topic?**

- People experiencing homelessness often die young and rarely access palliative care from specialists or other health care professionals
- The burden of looking after unwell people experiencing homelessness is often left to hostel staff
- Standalone training of hostel staff in palliative issues is not enough to increase palliative care access to this population

**What this paper adds?**

- In-reach support from one or two palliative care specialists, dedicating two half days per month each, can lead to a change in the hostel’s palliative care ethos by furthering hostel staffs’ understanding of palliative care and how it can benefit the homeless population
- This model upskilled hostel staff and empowered them to create more effective connections with external agencies, resulting in better support for their residents
- Due to the level of need, more training and support from palliative care and dedicated funded inclusion health primary care provision in this area is still needed

**Implications for practice, theory and policy**

- Palliative care specialists can have a key role in supporting a multidisciplinary holistic approach to the care of unwell people living within a homeless hostel
- To address health inequalities, palliative care specialists should work alongside hostel staff to explore ways to address the unmet palliative care needs of this marginalised population
- In light of COVID-19, remote support from homelessness champions and other professionals should be explored

## Introduction

On an average night, it is estimated more than 400,000 people living within the European Union, and more than 600,000 people living in America are homeless.^1^ Within Great Britain the number of people experiencing homelessness has been rising since 2010.^2^ Recent research demonstrated that within this population there are extremely high rates of multi-morbidity, frailty and age-related conditions at a young age.^3^ These factors contribute to the young average age of death for people experiencing homelessness.^4–6^ Palliative care involvement from specialists or other health care professionals for this population is rare for a number of reasons, including complexities in identifying who may benefit from palliative care, a lack of appropriate places of care for people with high care and support needs, mental health difficulties and/or addiction issues and the recovery focused nature of many homelessness services.^7,8^ Therefore in addition to often occurring at a young age, the deaths of many people experiencing homelessness frequently follow crisis-led hospital admissions.^9^ This lack of palliative care support and appropriate places of care means that burdens associated with supporting very unwell homeless people with complex problems, often fall on hostel staff; adequate support from health and social care providers is often lacking.^7,9^

Previous research highlighted a need for a more multidisciplinary approach when supporting people who are homeless and have complex and palliative care needs.^7^ In order to help hostel staff support residents with complex ill health, a 2-day educational training course on palliative care for hostel staff was developed and evaluated.^10^ The training increased the knowledge of hostel staff regarding how to access palliative care for residents and the awareness of signs of deteriorating health. However, due to high hostel staff turnover and lack of formal connection with outside agencies, it was concluded that for training to have a sustainable impact, it needs to be incorporated into regular support. This regular support could be driven by palliative care specialists. A palliative care approach is holistic, recognises that not everyone will recover, focuses on what ‘living well’ means to someone and can incorporate parallel planning (i.e. hoping for best, whilst planning for the worst). This is currently lacking within the recovery-focused homelessness services.

Studies have measured the impact of palliative and end of life support interventions for people experiencing homelessness including supporting people to complete advance care plans, supportive housing and harm-reduction services.^11^ These studies reported on a variety of outcomes such as increased numbers of advance care plans and referrals to palliative care services and were conducted in Canada, USA or Sweden. None provided direct palliative support to people living and working in hostels. Our project explored the challenges and benefits of embedding palliative care specialists, trained in homelessness issues, into UK hostels.

## Aim

To evaluate a model embedding palliative specialists, trained as ‘homelessness champions’, into hostels for two half-days a month to provide support to staff and residents and facilitate a multidisciplinary approach to care.

## Objectives

- Support and train palliative care specialists to become ‘homelessness champions’ to deliver training and support for hostel staff around signs of deteriorating health and access to support for people whose health is a concern.
- Support the champions to integrate person-centred multidisciplinary working into routine practice within hostels.
- Explore the perspectives of hostel staff and palliative specialists on the challenges and benefits of this model.

## Methods

### Design

An exploratory qualitative design was used with reporting guided by the Standards for Reporting Qualitative Research framework.^12^

### Participants and recruitment

Palliative care specialists (nurses and social workers) were recruited from hospices previously expressed an interest in supporting the homeless community during dissemination of our previous work.^7^

The role of ‘homelessness champion’ was incorporated into their job plan (i.e. voluntarily taking on this role within their established job). Two medium to high support-need hostels^13^ in the area served by the palliative care specialists were identified and approached by the researchers by email for potential inclusion in the project. Participating hostels identified one or two staff members interested in becoming link support workers to coordinate with the homelessness champions and facilitate meetings. Written informed consent was obtained from homelessness champions, hostel managers and staff.

### Procedure

**Figure 1.**
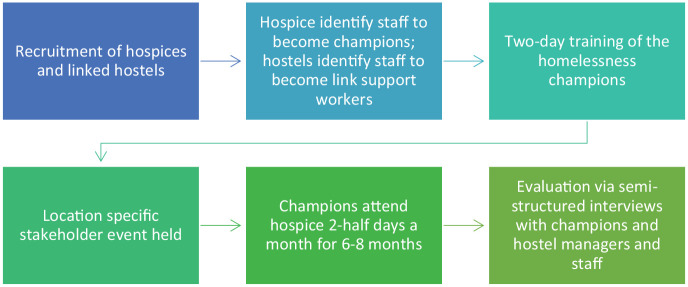
Flow diagram of project procedures.

Figure 1 outlines the steps that were undertaken in each area participating in the project. Table 1 outlines the role of the homelessness champions and the training they received.

**Table 1.** Role of the homelessness champions and the training they received.

Role	Training received
Aim: support hostel staff and residents for two half days a month each (incorporated into their job plan). The role was purposely kept fluid to enable them to adapt to the needs of the hostel	Aim: provide the knowledge, skills and resources needed to deliver training and support to hostel staff over a 2-day training course, led by three of the authors (CS, MA and NB)
Facilitating the integration of multidisciplinary working into routine practice to discuss residents of concern	Overview of the champion’s role, the homelessness landscape and integration of palliative care
Signposting and supporting referrals to external agencies, thereby increasing access to packages of care from social services and continuing health care funding	The nature, causes and consequences of homelessness, with an understanding of the contribution of adverse child experiences and complex trauma
Help hostel staff identify gaps and unmet need within the hostel	The complexity of need of people experiencing homelessness (physical and mental health difficulties often in association with addictions)
Offering to meet with and support residents of concern directly, where relevant. Support parallel planning *(i.e. ‘hoping for the best, planning for the worst’)*	Supporting people with complex needs within a hostel environment to access person centred care
Providing advice to hostel staff (individually or at team meetings) by delivering bespoke and responsive training to staff, including signposting to resources	Use of homelessness and palliative care toolkit (www.homelesspalliativecare.com), a resource pack containing presentation slides, case studies, lesson plans and activity sheets for use with hostel staff
Provide bereavement support to staff and residents	Awareness of the burden of death on staff and residents. Use examples of how different hostels respond to death of residents

#### Stakeholder event

To raise awareness of the project and obtain support from a range of services, location specific stakeholder events were held. People with lived experience of homelessness, frontline hostel and day centre staff, people working in palliative care services, commissioners and providers of primary care, mental health, drug and alcohol services and social care were invited. During this 2-hour event, details of the proposed project were presented followed by a discussion of how this could be adapted to the specific location. This meeting also served as a forum for links to be generated between different agencies.

#### Supervision and support

Throughout the project, homelessness champions were encouraged to contact (via phone or email) CS or MA to discuss the project and any concerns they had. CS is an inclusion health General Practitioner (GP) so could offer support and guidance including signposting for clinical issues. MA is a senior research fellow who provided support for any research-based queries.

### Data collection and analysis

#### Monthly reporting

To ensure impact and challenges were captured, the champions and the hostel link support workers were asked to provide monthly email or telephone updates to the research team. This included an overview of progress, including whether case review meetings were occurring, advice and training delivered or received, outline of support received by residents and referrals made. These updates were logged and stored in excel sheets.

#### Evaluation of project

Six to eight months (between December 2018 and June 2020) after the start of the project, individual semi-structured interviews were conducted by CS and MA with homelessness champions, hostel staff and hostel managers. The participants were informed the interviews formed the basis of the project’s evaluation and were conducted at their place of work. Interviews lasted between 60 and 120 min following an interview guide informed by an Expert by Experience. The interviews were audio recorded and transcribed verbatim. Thematic analysis guided by Braun and Clark’s^14^ framework was used to identify, analyse and report themes. Line-by-line coding was undertaken by MA in NVIVO 12^15^ and consensus was achieved through discussion between authors (MA, CS and BH). Higher level candidate themes were developed and discussed with a wider group of healthcare professionals, researchers and formerly homeless people.

### Ethics

Ethical approval for this research was granted from UCL Research Ethic Committee on 08/02/2019 [Project ID: 6927/002].

## Results

### Participant and hostel characteristics

Four hostels and two palliative care teams took part in this study (two hostels per palliative care team). No palliative care team or hostel approached declined to take part. One team identified two members of staff (two nurses) to become ‘homelessness champions’ and the other identified four (two nurses and two social workers). Participating hostels identified one or two members of staff to become the link support workers. Participant characteristics are outlined in Table 2 and hostels’ characteristics in Table 3.

**Table 2.** Participant characteristics.

Participant job	Hospice/hostel	Gender	Experience in years
Area manager	Hostel 1 and 2	Female	20+
Hostel support worker 1	Hostel 1	Female	16
Hostel manager 1	Hostel 2	Male	1
Hostel support worker 2	Hostel 2	Female	1
Hostel manager 2	Hostel 3	Female	1
Hostel support worker 3	Hostel 3	Female	18
Hostel support worker 4	Hostel 3	Male	0.5
Hostel manager 3	Hostel 4	Female	11
Hostel support worker 5	Hostel 4	Male	5
Homelessness champion 1: Nurse	Hospice 1 – linked with hostel 1	Female	8
Homelessness champion 2: Nurse	Hospice 1 – linked with hostel 2	Female	1
Homelessness champion 3: Nurse	Hospice 1 – linked with hostel 1	Female	2
Homelessness champion 4: Nurse	Hospice 2 – linked with hostel 3	Female	3
Homelessness champion 5: Social worker	Hospice 2 – linked with hostel 3	Female	5
Homelessness champion 6: Nurse	Hospice 2 – linked with hostel 4	Female	2
Homelessness champion 7: Social worker	Hospice 2 – linked with hostel 4	Female	7

**Table 3.** Hostel characteristics.

Hostel	Number of residents	Length of stay	Age of residents	Level of need	Dry or wet*	Food provided	No of deaths past year	Deaths throughout project	GP/nurse in reach**	Mental health in reach**	Drug and alcohol in reach**
1	35	Up to 2 years	22 – no upper limit	High support needs	Wet	Yes: three meals a day	5	0	In-reach GP who attends once a week	No	In process
2	58 (including 18 in bedsits)	6–15 months	18 – no upper limit	Medium to high support	Dry	Breakfast and dinner; no meals for bedsits	0	1	No, but good links with local homeless GP	No	No
3	60	Six months to 2 years	18–65	High complex needs	Wet. Not to drink in communal areas	No	3	3	No	No	No
4	50 plus 30 who have less support from the staff	18 months to 2 years	18–65	Medium complex needs	Wet. Not to drink in communal areas.	No	0 (1 died after move on)	2	No	No	In process

* Wet hostels allow alcohol to be consumed at the hostel where as dry hostels do not.

** ‘In-reach’ refers to a service going into the hostel on a regular basis.

### Monthly reporting, supervision and support

Information regarding residents with advanced ill health was collected monthly from the homelessness champions and the hostel link support workers has been summarised in Table 4. Data was returned to champions at the end of the project for verification. Early on, in one hostel, significant barriers to residents receiving adequate primary care were identified. MA, CS and one champion met with the practices to discuss the project and highlight the unmet needs of people within the hostels. These meetings explored challenges from hostel and healthcare providers’ perspectives along with potential ways to reduce barriers and improve collaborative working.

**Table 4.** Data collected monthly from the champions.

Hostel	Concerns raised and residents discussed	Advice and training	Progress
1	• Leg ulcers & sores on leg • Weight loss and malnutrition • Residents with cancer • Chronic obstructive pulmonary disease • Chest infection • Behavioural issues and poor memory following head injury • Hepatitis C • Chest pain • Resident assaulted by another resident • Reasons for leg amputation in resident (who was in hospital) • Admission of resident to hospital with septicaemia (infection from leg ulcers and incontinence)	• Advice given on numerous cases including encouragement to liaise with their GP where relevant • Advice regarding memory issues and advocating for referral to adult social care • Provided link and support to hostel staff to access training on dressing leg ulcers • Training around what palliative care is • Support to staff around resident adherence to their prescribed medication • Use of ‘Supportive and Palliative care Indicators tool 4 All’ (SPICT4All), which is a tool to help identify those with palliative care needs • Infection control	• Initially hard to engage with hostel staff and manager • Hostel staff became more engaged over time (3 months) • Regular dates of attendance for champion visit were then set and were well attended • Champion started attending GP meetings • Hostel staff concerns and GP concerns were shared • Integration of SPICT4All into regular resident assessments
2	• Chronic obstructive pulmonary disease • Safeguarding issues • Gastric cancer • Poorly engaging residents with hostel staff and external services • Leg ulcers • Hoarding • Lung cancer • Bereavement • Blood in vomit • Addictions	• Management of breathlessness – including use of fan, open windows • Advocacy around how to explore with residents what ‘living well’ meant to them • Teaching resident to apply dressings to his own leg (poorly engaging with nurse) • Alcohol related issues and impact • Liver failure advice • Discussion on who might benefit from palliative care • Overview of toolkit: Identifying residents of concern, liver disease map	• By the end, support from hostel staff was becoming more tailored to the needs of residents • Project encouraged hostel staff to follow up on residents – project link worker taking proactive lead on health / health advocacy • Successful death café (i.e., an event for people to get together to discuss death in a relaxed environment)^16^
3	• Hostel very concerned about a complex resident – incontinent, weight loss, unexplained bleeding, in and out of hospital. • Safeguarding issues in a resident suffering financial exploitation. • Death of a resident – huge impact on staff and residents	• Support around resident who was very unwell -spoke with GP and successful referral into hospice • Discussion around staff needing to balance a degree of risk-taking versus taking excessive control (this arose following an unwell resident losing autonomy and privacy due to staff concerns and anxiety) • Staff encouraged to complete risk assessments • Rights of residents under care act • Rights, safeguarding and duty of care • Importance of occupational therapist, GP, social services and their roles • Capacity assessment • Developing care plans for residents – person centred approach • Overview of homeless palliative care toolkit • Laws and advocacy for disabled people • Referral process into hostel explored – supporting manager to say no if needs were too great • Bereavement support	• Resident who had caused huge concern was supported compassionately and given back more control • Staff less anxious about resident • Staff realized not responsible for everything • Staff now scrutinising referrals into the hostel to ensure they are able to provide appropriate support • Supported staff to challenge a negative decision following a social services care act assessment - resulting in residential placement for resident due to his high care and support needs
4	• Very unwell resident with complex needs, addictions, and human immunodeficiency virus (HIV) • Fluctuating engagement from a number of residents around their health • Malnutrition • Resident going ‘missing’ for long periods of time • Concern around calling 999 (i.e., the UK emergency number) when resident does not want this, and support workers feel they have inadequate information/knowledge on which to base decisions • Staff bereavement and concern for residents experience of loss when other residents die • Mental health crises, dealing with suicidal ideation and engagement of community mental health service in the context of addiction	• Direct work with GP • Education around liver disease progression • Worked directly with drug and alcohol service to support chaotic resident • Met with resident to discuss their wishes • Met with family member to discuss their concerns about a resident with palliative care needs • Referral to palliative care • Palliative care • Mapping tool, red flags, surprise question, liver map, residents of concern and toolkit in general • Discussion around Mental capacity Act and assessments	• Building relationships with staff • Some residents beginning to engage with champions • Bereavement support to staff • Organised a hospital bed for resident • Worked with support worker to enhance regular adherence to nutritional drinks for resident with malnutrition

## Themes

Thematic analysis of semi-structured interviews exploring the impact of this model identified five main themes (see Table 5).

**Table 5.** Themes and subthemes.

Themes	Subthemes
Impressions of the model	Needed and valuable model
	Benefits of the stakeholder meeting
Developing partnership working between hostels and champions	The process of partnership development
	Development of case management meetings
Developing a holistic palliative ethos	Understanding the benefits of palliative care
	Death café and vigil
Improvement of hostel and external service working	Hostel staff empowered
	More external service support
Challenges	Time and organisational barriers
	Primary care barriers

### Impressions of the model

#### Needed and valuable model

The champions were shocked at the level of need within hostels and the lack of equitable access to high quality care. They believed there was clear need for this role to continue

“*I would like to see it embedded. I would like to see us continue working with the hostels. Having that link. We link in with the nursing homes . . .to support them. So having that role for hostels as well especially like (Hostel 1) where I think there are more [sick residents] there and there is the need*”

Homelessness champion 1

Similarly, hostel staff felt the project was extremely beneficial as they move from feeling isolated and working on them own to feeling supported and understood:

*“Beneficial doesn’t really sum it up. . .invaluable. Because we have been working in isolation for such a long time and people don’t really know how hard it is to work here. It is just a shame that this hasn’t always been in place”* Hostel manager 2

The homelessness champions highlighted that being palliative care specialists meant they approached the residents’ care holistically, understanding how various factors interlinked:

*“I suppose having worked in other areas of healthcare, the palliative care model is very much one of . . . physical, psychological, social, spiritual, financial. That’s kind of our bread and butter everyday working. . .We tend to meet people, mostly, with the physical need being the priority but we’ve got an awareness of how important it is to get those other bits sorted. That’s kind of a nice basis to start with I suppose.”* Homelessness champion 3

Having both a palliative care nurse and social worker working together was a further advantage in comprehensively addressing the needs of the staff and residents:

*“I don’t know whether I would have been as confident doing it without that [combination of a social worker and nurse]. It’s been so good to work together through the whole thing because there’s been such a holistic health and social need within there. It has been complementary.”* Homelessness champion 4

#### Benefits of the stakeholder meeting

Starting with the stakeholder event was deemed helpful, as it increased people’s knowledge about palliative care and how it’s principles could support this population.

“*. . . . I think that everybody came there thinking that they knew about palliative care. But I think by the time it ended, there was a very different perception of palliative care. . ., I think that had quite a big impact. And I think the questions that came afterwards, you could see that you had got people thinking, and I think that’s really important. . .And I think a lot of the people there, they got it. They got it. I think that part of that was the reason that they were so keen*.” Hostel area manager

The managers were impressed with the level of interest, concern and commitment expressed by the range of professionals attending the stakeholders event. These were often the first time individuals from this range of organisations had gathered to discuss this population:

*“I think that one of the very first things that I was overwhelmed with was. . . . I could not get over the amount of interest that came from other external stakeholders. They were definitely committed. They wanted to be involved in the project. They were going to do anything to support us. To me. . .it was one of the things that really excited me.”* Hostel area manager

The stakeholder event was seen by some champions as important to help validate their role and help with the development of the trust and partnership between them and hostel staff:

*“The stakeholder meeting, I think, was invaluable for them to understand that we were not trying to trick them or implicate them in any way in what they’re doing, but to just support them.”* Homelessness champion 4

### Developing partnership working between hostels and champions

#### The process of partnership development

The development of partnerships between the homelessness champions and the hostel staff was key to the project’s success. Hostel staffs’ experiences with external services had often previously been negative and critical. They were consequently fearful of being judged. Champions had to breakdown these barriers by developing trust as well as demonstrating the value they could bring to staff and residents.

“*When we were initially approached, we were like “oh god, more work, what else are they going to throw at us?”. . .And it was a little bit like airing our dirty laundry, because we were a bit scared that there were things we were doing wrong, and that they were going to bring them to the forefront with other professionals, and then how would we be viewed as well. So there was an element of that as well but that hasn’t been the experience, it’s been the opposite*.” Hostel manager 2

Champions described how important it was to not have pre-conceived ideas about what was needed and the importance of learning together and fostering a collaborative approach.

*“I wasn’t sure whether I was going to be accepted there. I didn’t really want to go there and say “ok I’m the trainer, I’m the champion, I’m here to train you”. I really didn’t want to have that approach; I was really keen on . . .lets learn together”* Homelessness champion 7

In one of the hostels, the champions were called in to help before they were scheduled to begin, due to a crisis with a resident. Though less planned, this resulted in the champions quickly gaining trust and a successful partnership through demonstrating the support they could bring to the staff and the residents.

*“I think the crisis that brought us into the project early. That really helped show everybody what we’re working for. I think the staff at that point, really saw the value of having us there, and we really saw the need, because it was pretty disastrous, and the ongoing issues with the GP surgery and what have you, but I think the good that came of that was the foundation of the relationship that we managed to establish. . .”* Homelessness champion 4

In another hostel, it took time to develop the partnerships, due to a lack of faith in what external services could offer combined with burnout.,

*“I feel like the first time I went in and met the staff, there was one that was asleep, one just didn’t say a word. No one was looking at you, no one seemed interested, at all. . . .so my kind of initial work with the staff was just “who are you? What do you do? How do you like your job?” and just trying to build up a relationship to try and then get them to open up.”* Homelessness champion 3

As their relationship developed, the champions provided emotional support to the hostel staff. The quote below was following the death of a resident but the emotional support was valued in many other situations also:

*“It was just to sit with somebody and not talk about the day to day working, but just like how are you feeling about this as a person, how has this affected you, and letting her cry and feel sad about it.”* Homelessness champion 4

#### Development of case management meetings

The project resulted in the development of regular case management meetings. During these meetings staff and champions discussed residents they were concerned about, explored barriers to accessing support and possible solutions. These meetings provided the hostel staff with support, advice, and a plan on how to approach the issues residents were facing.

*“We just talked through what the current concerns were and what the staff concerns were and how particularly for one of the residents, how we just don’t know what else to do with her now. [The champion] was able to come and give some advice on the spot and just say, “Well, have we tried this, have we tried that?” Hostel manager 3*

### Developing a holistic palliative ethos

#### Understanding the benefits of palliative care

Initially, some hostel staff were concerned that the project, as it was around palliative care, would focus solely on death. To overcome this, champions provided reassurance and clarity about how they might provide support.

*“It’s not just about someone dying. It’s someone who can become quite poorly, how to manage their health. There’s a lot more. Initially, from what we understood, it’s for people who die. Then we saw, “Hang on, we only have one death every few years. We don’t need to be part of this pilot.” We had that impression. When [the champion] came in and then we did all this other work, we were, “No, it’s actually not just about death. It’s other things that relate to death or can cause death, or some things about welfare.”* Hostel manager 1

The champions witnessed a shift in the mind-set of most staff from the hostel. Initially, most hostel staff would not contemplate a hostel being an appropriate place of death. However, as the project continued there was a growing acceptance that if residents saw the hostel as their home and wished to die there, if support was in place, that would be the right thing to aim for.

*“. .initially when we think that someone is going to die, we would have said “no, no, no, we need to move them on quickly, we don’t want them dying within the hostel” . .but then we started to change our way of thinking because of this. . .we did start saying, well yes this is his home” Hostel m*anager 2

The hostel staff saw the importance of taking a more person-centred approach to care, with the needs and wishes of the residents being listened to and incorporated into planning and discussions.

*“The critical reflection, the thinking about how to communicate differently on what the barriers might be between the conflicting goals of the resident and the service. I think managements really moved forward on that.”* Homelessness champion 5

#### Death café and vigil

In one of the hostels, a psychologist linked to the palliative care specialist’s team ran a death café with the hostel key link worker and champion. The death café helped to address some of the anxieties that staff and residents had around talking about death. This left them more open to having conversations around death, dying and people’s wishes. There are plans for this to become a regular occurrence at the hostel:

*“The death café was something when I heard I thought “oh my god, what is this?” I would love the death café to be an ongoing part of the work that we do. The feedback from the death café would, again, bring somebody to tears. . . .Because up until then there was a real fear, real fear for staff to perhaps engage in talking about death and what that might look like, or preparing for it. And the death café was probably one of the biggest breaking down of barriers. They actually saw that these residents wanted to talk about it.”* Area manager

Following the death café, the manager realised the impact that deaths had on the residents and staff. Instead of believing the best thing to do is to move on quickly when a death of a resident occurs, the hostel held a vigil to acknowledge and celebrate the life of the resident who passed:

“*Previously when we have a death, you do all the paperwork, you do the incident report, and then get the coroner’s report. That’s just the general paperwork stuff. For this guy, we did something special. We did a little vigil. We had little pictures cut out for him. We had these little cards made for him. We had it in the canteen. His friends came and that little service happened. . . .”* Hostel manager 1

This openness and engagement around death, dying and memorials was received by hostel residents positively with some indicating that they felt cared for by hostel staff:

*“What some people said following on that from the service, “You guys actually care.” Naturally, people think, “We’re just a number in a hostel. You want us out in 12 months. The fact that you did the service shows you guys actually care about us.”. .”* Hostel manager 1

The new approach hostels were taking to life, death and bereavement, meant a shift occurred from residents blaming staff for deaths occurring, to grieving and supporting each other:

*“Because before tenants would kind of. . .blame staff if someone died in the project. A lot of the tenants will now be part of the caring, or they will pop in [to vigils]*.” Area manager

### Improvements in hostel and external service working

#### Hostel staff empowered

With increased knowledge and the support of the champions, hostel staff felt more empowered to work with and challenge external services where necessary. This included proactively contacting the relevant services, chasing up referrals and feeling confident that they knew the needs of the residents and when services should be involved:

*“I think everyone is just a little bit braver now, to step forward and [to outside services] be like, actually, this is how it is supposed to be. You’re not supposed to be telling us that.” Hostel* support worker 3

#### More support from external services

This empowerment led to the development of better relationships with external services that could support hostel residents:

*“I think it’s [the project] helped us make contact with other professionals. So not just sitting on the fence waiting for them. It made us be a bit more proactive. And to not be so scared to make some of those key contacts. . . .we have definitely built up better relationships with some hospital staff and our local pharmacies, places like that.”* Hostel manager 2

In the hostel that had GP in-reach already, the relationship between the GP and hostel staff improved greatly to now communicating and working together to provide residents with the best care possible.:

*“I’m really pleased with the fact that when I went in, the GP would go “oh, the staff there can be really difficult”. And the staff would go “oh, that GP, she doesn’t listen to us, she doesn’t care”. And changing that to them sitting in the same room as the GP talking about a patient that had just finished their consultation. . to make a plan going forwards to make sure that he was looked after. And I was just like that’s really. . .I feel like I’ve done that, and that’s a really good thing to have that relationship.”* Homelessness champion 3

In addition, the champions felt they had a role in educating and changing the values and inclusiveness of their own palliative care service to improve the partnership between the hostel and palliative care teams:

*“I think bringing out of the learning we’ve had as well and bringing that back to our own service and other services. Just talking about this service user group. I’m proud of that to be able to say, we’ve got that knowledge around this, it’s not just talking.”* Homelessness champion 5

Through developing trust with the champions, residents who had previously struggled to engage with other services, began to engage:

*“I think she [resident] gets that the help is out there and actually it’s time to accept some help. She does accept some help.”* Hostel support worker 5

### Challenges

#### Time and organisational barriers

One champion felt the project was not sustainable unless there was to be more time allocated to it. Whilst their line managers were very supportive, they were unable to protect champions’ time for the project or to reduce their caseload:

“*It’s not sustainable and I think if this is something that is going to carry on indefinitely, I don’t think it’s going to work. Being quite cynical about it or pragmatic about it, I think, it’s just not enough time. . .it’s at risk of burning us all out. I’m cramming in my normal work into smaller amounts of time and potentially doing less of a thorough good job because I’m responding to emails, making phone calls, and mentally my head’s in that project.”* Homelessness champion 4

The champions felt that with more time they would be able to achieve much more including being more proactive so that all residents could have care plans and access increased support:

“*I suppose if we have more time, we’d be able to go through all the patients with them that are complex and causing them stress. We’d be able to come up with plans for all of them. We’d be able to review their policies and look through what exactly it is that they’re worried about or what isn’t in place. We’d be able to, I suppose, provide more general advice around health and social care and create lasting documents or procedures . . .”* Homelessness champion 4

The hostel staff were also challenged by a lack of time and unpredictability in their work. To address this the champions needed to be flexible. Occasionally they would arrive at the hostel and staff or residents were not able to meet with them. To deal with this, they needed backup plans of things they could do if residents or staff are unavailable, such as reading through policy documents or seeing another resident.

“*I think timing has been difficult because it’s quite often– [the champion] might make an appointment to come to see a resident, she might arrive, the resident’s not here. She might come and see us and then something’s happened and we’re not available because this project is so busy. I’d really like to be able to say, “Let’s concentrate on this for today and that for tomorrow,” but it just doesn’t work like that*.” Hostel manager 3

#### Primary care barriers

Some hostels were well supported by GPs providing regular in-reach clinics. However, in one hostel in particular, the champions described feeling shocked by the response of some of the GPs who demonstrated a lack of compassion and understanding for the population and their needs. Champions and hostel staff struggled to get referrals to palliative or mental health services and adequate support for residents who were unwell. This made the role of the champion even more important, but also more challenging.

“*. . .you’ve got GPs that are now refusing to go into the hostels at all and are refusing to see any homeless patient. . .[GPs] are insisting on having . . .somebody in attendance with them [during appointments] because they don’t trust them for whatever reason to either retain the information or be a capable enough human being to sit and take in information. It’s so dehumanizing that you’ve got these people with such power, literally over your life and death, treating you like you’re nothing, [implying] “You’re not worth my time.”“* Homelessness champion 4

## Discussion

### Main findings

This project is the first to evaluate a model providing in-reach support from palliative care professionals into homeless hostels. Overwhelmingly the champions and hostel staff felt this model was beneficial for the staff and residents. Despite embedding palliative care specialists (nurses or social workers) for only two half-days a month each, the champions developed trusting partnerships with hostel staff and created an ethos more open to a palliative approach, which was more person-centred care and explored residents’ wishes and insights. Previous research has shown hostel staff are reluctant to discuss palliative care with residents, but using a parallel planning approach (‘*hoping for the best while planning for the worst*’) can be helpful in improving communication for those whose health may be deteriorating.^16^ The champions of this project were able to start de-stigmatising death and dying. This was evidenced by the perceived success of vigils and death cafés, and a planned death within a hostel.

A palliative approach was found to be helpful in supporting staff to take a more person-centred approach to care and to recognise when more health or social care input was needed Hostel staff were empowered by their increased knowledge and understanding, which enabled them to advocate for what their residents required from external services. Hostel staff became to recognise where their own responsibility began and ended and the steps they could take to maintain these boundaries. In turn the relationships between hostels and external agencies began to improve. Champions also provided emotional support to hostel staff and residents around bereavement.

A barrier some champions faced was the lack of engagement of some GPs with this population. Previous research has highlighted the barriers people experiencing homelessness face accessing healthcare^17^ and our research highlights an urgent need for more equitable access to high quality primary care. In the two hostels that did not have an in-reach GP or nurse or close links to primary care, the unmet needs of the hostel staff and residents was greater, and champions filled gaps that should have been provided by primary care. Work is ongoing to reduce health and social care barriers, such as by the charity Groundswell^18^ who developed a pocket-sized laminated card explaining people’s rights to access primary health care and peer support for GP registration and appointments. However, more training and support or dedicated funded inclusion health primary care provision in this area is still needed. Another challenge for the champions was the lack of time they had at the hostel. This project has shown how much can be achieved with as little as two half-days a month per champion but going forward a funded role would allow for more time to be dedicated towards people experiencing homelessness.

### Strengths and limitations

This model provided and evaluated the knowledge, tools and access to support of a population group that seldom receives palliative care.^19^ In-depth interviews gave us rich data and allowed us to explore the impact of the model. However, we were unable to collect post-intervention quantitative data, in the form of staff questionnaires, due to high staff turnover so are unable to quantify the impact of the project on hostel staff morale or hostel residents’ quality of life. The interviews were conducted by the authors, and good rapport had been established and allowed the researchers and participants to discuss sensitive subjects freely. However, the fact this relationship may have led to some positive bias in participants’ responses. Despite this, participants were forthcoming in the challenges they faced.

### Direction for future research

This project was conducted prior to the COVID-19 pandemic. Since the pandemic, the champions have continued to support the hostels both in person and remotely. Future research should explore remote learning, training and support. The stakeholder event appeared a helpful way of bringing a range of professionals together. Other research has demonstrated how stakeholder events are beneficial in creating opportunities and sharing knowledge.^20^ Future research could explore the impact of holding stakeholder events as a way of addressing the needs of people experiencing homelessness by creating links between the services needed to address the complex needs of this population. A similar model exploring the benefits of embedding inclusion health professionals, particularly for hostels with a lack of primary care support, should be explored. It is also important to ensure the voices of people experiencing homelessness are heard and the impact this model has on them should be explored once the model has been embedded long-term.

### Clinical implications

Given the reported barriers to accessing palliative care for people who lack capacity and surrogates,^21^ integrating palliative care specialists within homeless hostels, as this model proposes, is valuable. Palliative care communities should reach out to local hostels to explore how best to support staff and residents. The expertise in inclusion health of the principal investigator in this project is likely to have impacted on this model’s success. Any palliative care team and hostel looking at applying this model should have someone experienced in inclusion health as part of the team, to support staff in dealing with complex issues.

## Conclusion

This study is the first of its kind to evaluate a model of embedding palliative care specialists into homeless hostels. It highlighted the huge inequity people experiencing homelessness face and successfully demonstrated the benefit of joined up support for both hostel staff and residents. It goes some way towards addressing the aspiration that people experiencing homelessness should live and die with dignity and respect.
